# Partners in Crime: Beta-Cells and Autoimmune Responses Complicit in Type 1 Diabetes Pathogenesis

**DOI:** 10.3389/fimmu.2021.756548

**Published:** 2021-10-07

**Authors:** Eliana Toren, KaLia S. Burnette, Ronadip R. Banerjee, Chad S. Hunter, Hubert M. Tse

**Affiliations:** ^1^Department of Medicine, Division of Endocrinology Diabetes and Metabolism, University of Alabama at Birmingham, Birmingham, AL, United States; ^2^Comprehensive Diabetes Center, University of Alabama at Birmingham, Birmingham, AL, United States; ^3^Department of Microbiology, University of Alabama at Birmingham, Birmingham, AL, United States; ^4^Division of Endocrinology, Department of Medicine, Johns Hopkins University School of Medicine, Baltimore, MD, United States

**Keywords:** beta-cell, beta-cell heterogeneity, pancreatic islet, autoimmunity, ER stress, oxidative stress, Type 1 Diabetes

## Abstract

Type 1 diabetes (T1D) is an autoimmune disease characterized by autoreactive T cell-mediated destruction of insulin-producing pancreatic beta-cells. Loss of beta-cells leads to insulin insufficiency and hyperglycemia, with patients eventually requiring lifelong insulin therapy to maintain normal glycemic control. Since T1D has been historically defined as a disease of immune system dysregulation, there has been little focus on the state and response of beta-cells and how they may also contribute to their own demise. Major hurdles to identifying a cure for T1D include a limited understanding of disease etiology and how functional and transcriptional beta-cell heterogeneity may be involved in disease progression. Recent studies indicate that the beta-cell response is not simply a passive aspect of T1D pathogenesis, but rather an interplay between the beta-cell and the immune system actively contributing to disease. Here, we comprehensively review the current literature describing beta-cell vulnerability, heterogeneity, and contributions to pathophysiology of T1D, how these responses are influenced by autoimmunity, and describe pathways that can potentially be exploited to delay T1D.

## Introduction

Type 1 diabetes (T1D) is a chronic autoimmune disease in which loss of beta-cell mass and subsequent insulin-insufficiency leads to hyperglycemia. T1D is linked to secondary complications including cardiovascular disease, kidney disease, and neuropathy (1). T1D is the most common form of diabetes in children, comprising approximately 75% of new diabetes diagnoses in patients under 19 years of age (2). Nonetheless, T1D is not a disease only of the young. Epidemiological studies now show that the incidence of autoimmune diabetes in adults (age 30 – 49 years) is at least as high as young adults (age 15 – 19 years) (3). Incidence of T1D is between 4 and 41 per 100,000 in the United States, but interestingly there is significant geographic variation in incidence rates worldwide. Asian countries have relatively lower rates of T1D, while Switzerland, Finland, Norway, the UK, and Sardinia have among the highest rates, with values greater than 20 per 100,000 people (4). Even considering the large geographic variability, overall new diagnoses are on the rise in both childhood and adult populations.

T1D is a multifactorial disease; both genetic and environmental factors contribute to risk. While incompletely understood, putative environmental triggers include microbial infections, neonatal nutrition status/weight, and exposure to certain toxins, such as nitrates (5). Following a triggering event in genetically-susceptible individuals, immune effector cells infiltrate the pancreas and activate inflammatory pathways to mediate targeted destruction of insulin-producing beta-cells. Since the early 1970’s, when the genetic connection between human leukocyte antigen (HLA) and T1D was first discovered, pathogenesis of T1D was largely defined by autoimmunity and the selective presentation of islet autoantigens. Strictly defining T1D by an immunological mechanism, however, does not acknowledge any potential role for the beta-cell itself in promoting disease pathology. Mounting evidence indicates the beta-cell is more than just a passive target in the development of T1D: the lack of long-term success with immune intervention therapies, the existence of islet autoimmunity without T1D development, and the persistence of beta-cells after diagnosis and T1D progression, all provide evidence that the beta-cell is an active participant along with the immune system in T1D pathogenesis (6, 7).

In this review, we will focus on the beta-cell in both healthy and T1D environments. We will explore inherent beta-cell heterogeneity and vulnerabilities, contributions to the local inflammatory environment, and how the beta-cell response to metabolic stress can perpetuate disease. Shifting focus from the beta-cell as a passive target to an active participant in disease progression will help identify novel therapeutic approaches, potentially leveraging these unique beta-cell responses and susceptibilities for both treatment and prevention of T1D.

## The Beta-Cell: Characteristics That Impart Vulnerability

**Figure 1:**
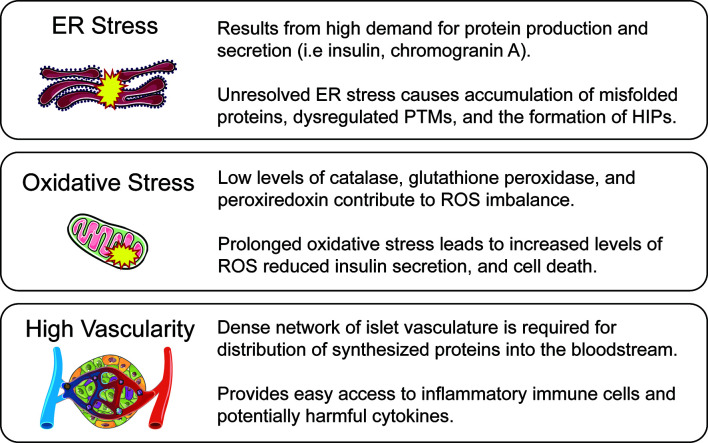
Beta-cell vulnerabilities. While autoimmunity is a major driver of T1D pathogenesis, innate features of beta-cell biology make it a complicit partner in disease progression. These beta-cell characteristics are a result of normal beta-cell function while also active contributors to disease amplification. ER stress is caused by the high protein production and secretory demand of the beta-cell, but in excess leads to misfolded protein response and the generation of HIPs through PTMs. Oxidative stress is caused by an imbalance between the generation of ROS and their detoxification by antioxidants. The reduced antioxidant capabilities of the beta-cell can lead to impaired function and cell death. A densely vascularized environment is required for secretion of insulin and other peptides directly into the bloodstream, but creates a direct dialogue between the beta-cell and potentially harmful immune cells and inflammatory cytokines which may further lead the cell toward stress and apoptosis.

In 1985, Dr. Gian Franco Bottazzo’s lecture titled “Death of a Beta Cell: Homicide or Suicide?” posed the idea of beta-cell fragility (8). Dr. Bottazzo questioned whether beta-cells were innocent bystanders of immune attack or contributors to their own destruction (9). Beta-cells must rapidly respond to glucose fluctuations by secreting the appropriate amount of insulin to maintain euglycemia, a taxing process that, even in healthy cells, makes them vulnerable to stressors such as inflammation and nutrition excess. The metabolic demand associated with tightly regulated insulin secretion, paired with a highly vascularized environment, reduced antioxidant defense mechanisms, and sensitivity to proinflammatory cytokines, makes beta-cells uniquely susceptible to autoimmune-mediated destruction (**Figure 1**).

### Secretory Demand

**Figure 2:**
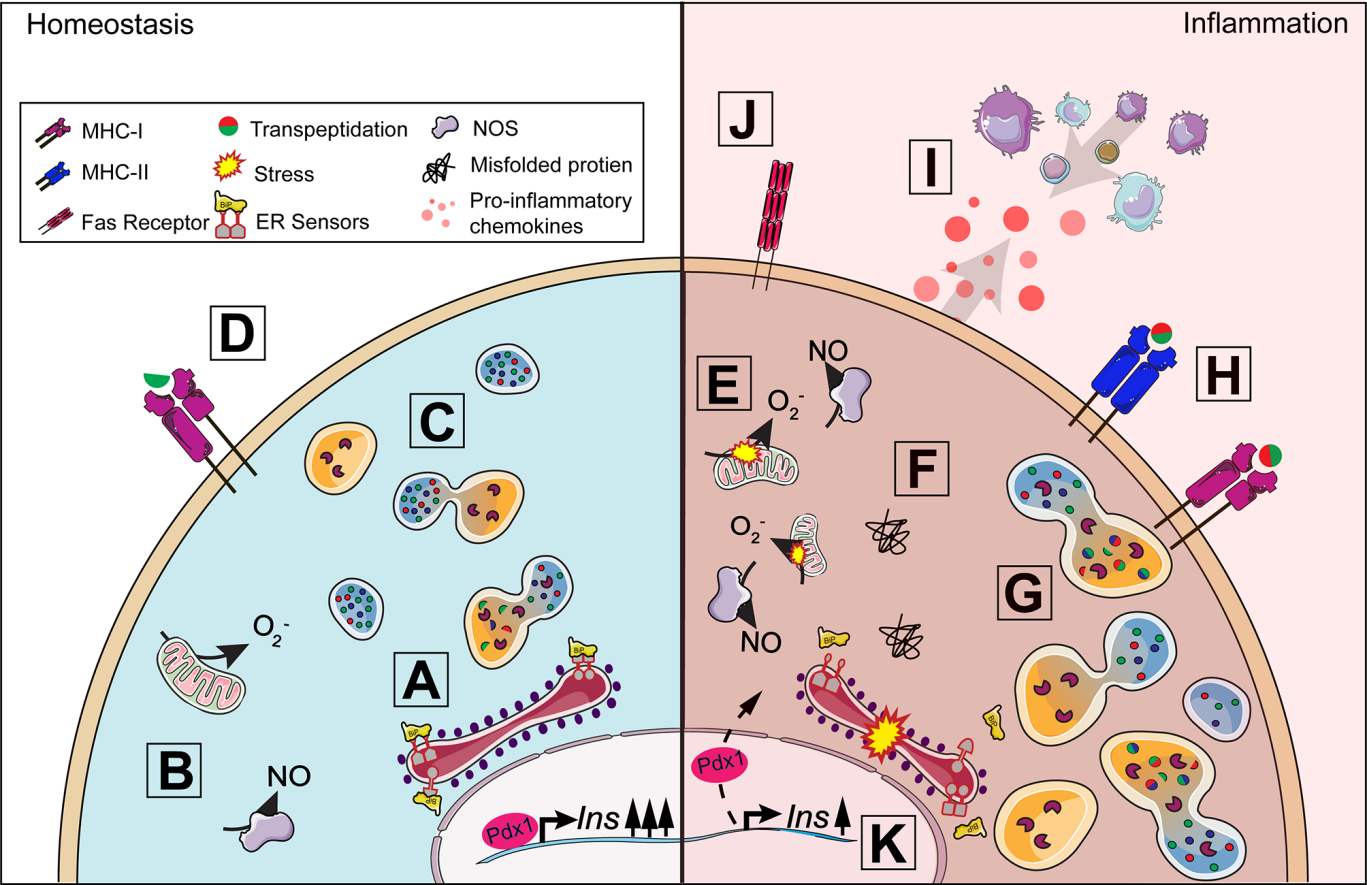
Beta-cell response to inflammation. Under homeostasis conditions, insulin production is tightly coupled with cellular metabolism including protein synthesis in the endoplasmic reticulum (ER) **(A)** and mitochondrial function **(B)**. When insulin secretory granule proteins are in excess, they can be broken down and recycled by crinophagy, a process by which granules fuse with lysosomes **(C)**. Some peptides from this degradation process are presented on MHC-I **(D)** and, in healthy cells, should not lead to activation. A proinflammatory environment around the islet exacerbates ER and oxidative stress **(E)** contributing to the dysregulation of multiple processes in the beta-cell. The accumulation of misfolded proteins can result in the activation of the unfolded protein response **(F)** and increase lysosomal degradation of insulin secretory granule proteins **(G)**. Protein degradation under stress can lead to the production of neo-antigens, such as hybrid insulin peptides, through transpeptidation. ER and oxidative stress results in the upregulation of MHC-I and the unique expression of MHC-II **(H)** by the beta-cell allowing for increased presentation of potential neo-antigens to T cells. Fas receptor expression **(J)** makes the beta-cell vulnerable to Fas-mediated apoptosis. The release of chemokines **(I)** from the beta-cell further contributes to immune cell recruitment and the development of insulitis. Insulin production can be affected as disturbances in cellular homeostasis can lead to the translocation of Pdx1 from the nucleus to the cytoplasm, decreasing insulin production **(K)**.

The beta-cell is responsible for producing and secreting multiple secretory granule proteins including insulin, chromogranin-A (ChgA), and islet amyloid polypeptide (IAPP). The endoplasmic reticulum (ER) is the site of protein production and relies heavily on Ca^2+^ concentrations to maintain the environment needed for proper protein synthesis and folding (**Figure 2A**) (10). Insulin secretory demand makes the beta-cell particularly vulnerable to exceeding ER protein folding capacity, which leads to the accumulation of misfolded proteins and a disruption of ER homeostasis (**Figure 2F**) (11). This physiological state is termed ER stress (12). Prolonged efforts by the cell to correct misfolded proteins can lead to unregulated changes in enzyme activity, reduced beta-cell function, and induction of apoptosis (13–16). To meet the metabolic demands of glucose-stimulated insulin secretion (GSIS), the beta-cell requires a tightly-coupled process with cellular metabolism to properly maintain euglycemia (17). In brief, glucose is transported into the beta-cell *via* the glucose transporter 2 (Glut2) in rodents (GLUT1 and 3 in humans), converted to pyruvate, and shuttled into the mitochondria where it is used for ATP production (18). Changes in the ATP to ADP ratio lead to beta-cell depolarization, Ca^2+^ influx, and insulin release (19–21). *Insulin* mRNA is translated at the ER following nutrient stimulation, which in rodents can signal up to a 10-fold increase in insulin synthesis at a rate of 1 million molecules per minute (22, 23).

To meet these high demands, beta-cells have an extensive ER with multiple chaperones to aid in protein folding, packaging, and secretion. However, high protein synthesis puts a significant amount of stress on the ER. The unfolded protein response (UPR) is triggered when an excessive amount of misfolded proteins accumulate in the ER, which can be caused by overnutrition, increased reactive oxygen species (ROS), or proinflammatory cytokines (24, 25). Three major sensors of the UPR are protein kinase RNA-like endoplasmic reticulum kinase (PERK), inositol-requiring enzyme 1 alpha (IRE1α) and activating transcription factor 6 (ATF6) (13, 26). In an unstressed state, these sensors are bound to the ER chaperone binding immunoglobulin protein (BiP) (**Figure 2A**). Accumulation of misfolded proteins leads to the dissociation of BiP from the three UPR sensors (**Figure 2F**) (27, 28). Together, the UPR sensors alleviate ER stress by attenuating global protein synthesis to reduce the load of unfolded proteins, increasing chaperone synthesis to guide protein degradation or refolding misfolded proteins, and synthesizing lipids to increase ER volume (29).

In addition to protein synthesis, the ER is also responsible for storage of intracellular Ca^2+^ and therefore, regulates calcium-dependent signaling within the cell, such as protein folding and enzymatic function (13). ER stress disrupts intracellular Ca^2+^ balance influencing multiple processes, including activation of cytosolic post-translational modification (PTM) enzymes by facilitating their translocation into subcellular compartments. This imparts downstream changes in gene expression, protein conformation, and enzyme activity (30–33). Dysregulation of PTM enzymes has been linked to the development of rheumatoid arthritis, celiac disease, and T1D (34–38). In T1D, this includes citrullinating peptidyl arginine deiminase (PAD) enzymes and tissue transglutaminase 2 (tTG2) deaminating enzyme (13, 39). PAD, tTG2, and similar enzymes can alter the binding affinity of peptide epitopes, such as insulin, to major histocompatibility complex (MHC) class II, resulting in increased CD4 T cell activation (36, 40). Inhibition of systemic PAD enzymes in NOD mice can protect against diabetes progression, suggesting a role in T1D initiation (41).

Stress-induced PTMs can also result in the creation of neo-antigens in peripheral tissues for which the thymus has not established tolerance. Many T1D neo-antigens generated from PTMs have been identified (42). Some PTMs can lead to non-functional protein products resulting from alternately spliced RNA called defective ribosomal products (DRiPs) (43). Increased expression of DRiPs from insulin have been measured in beta-cells in response to ER stress and can be recognized by T cells from patients with T1D (44, 45). Hybrid insulin peptides (HIPs) are another group of neo-antigens generated from transpeptidation, a PTM where insulin peptides are covalently linked to other beta-cell granule peptides including insulin C-peptide, IAPP, and ChgA (**Figure 2G****)** (46, 47). HIPs are not only recognized by autoreactive CD4 T cells in mouse models of T1D, but CD4^+^ T cells from patients with T1D recognized HIPs as well, signifying their potential role in disease initiation and progression (48–50). Our understanding of how neo-antigens are generated and contribute to the development of autoreactivity in T1D is currently unknown. Future studies are warranted to further define how ER stress and subsequent downstream disruptions induced by the secretory demands of the beta-cell can influence autoreactive T cell responses and beta-cell vulnerability in T1D (**Figure 1**).

### Oxidative Stress

Oxidative stress occurs when there is an imbalance between ROS generation and antioxidant activity (51). Superoxide is primarily a byproduct of normal cellular metabolism that is generated in the mitochondria and cytoplasm (**Figure 2B**) and is an initiating free radical that can result in the formation of other reactive species such as hydrogen peroxide (H_2_O_2_), hydroxyl radical, and peroxynitrite (52). Free radicals are highly reactive and can induce cellular damage, but antioxidants including superoxide dismutase (SOD), catalase, glutathione peroxidase (GPx), peroxiredoxins, thioredoxin, and glutathione protect the cell by detoxifying these reactive species (53, 54). SOD dismutates superoxide to molecular oxygen and H_2_O_2_, a less destructive oxidant and signaling molecule. H_2_O_2_ regulates insulin secretion by activating the second messenger c-Jun N-terminal Kinase (JNK) (55). This leads to decreased insulin production through the translocation of the transcription factor pancreatic and duodenal homeobox 1 (Pdx1) from the nucleus to the cytoplasm, resulting in decreased *Insulin* transcription (56) (**Figure 2K**). H_2_O_2_ is further converted to oxygen and water by catalase, GPx, and peroxiredoxin. Increased levels of H_2_O_2_ can form extremely reactive hydroxyl radicals through Fenton reactions with free iron present in the cytoplasm, which has downstream negative effects on intracellular calcium levels, protein synthesis, glycosylation, and redox status (53). Beta-cells, however, have decreased antioxidant levels and therefore, are highly susceptible to free radical-mediated damage (**Figure 2E**). Rodent and human beta-cells have reduced transcriptional and protein levels of cytosolic copper/zinc (Cu/Zn) SOD1, mitochondrial manganese (Mn) SOD2, catalase, and GPx, which can result in exacerbated levels of superoxide, H_2_O_2_, hydroxyl radical, and peroxynitrite that are implicated in beta-cell death in T1D (57–61). The inability to properly restore cellular homeostasis due to the negative effects of oxidative and ER stress can induce apoptosis in insulin-secreting beta-cells. Increased beta-cell apoptosis has been measured in patients with T1D and NOD mice when compared to healthy controls (14, 62–66). In addition to oxidative stress, the beta-cell is also impacted by the islet microenvironment in which it is closely associated.

### Islet Vascularization and Exposure to Cytokines

Importantly for T1D pathology, islets are highly vascularized. This provides an interface by which immune cells, even from distant sites, can gain local access to pancreatic islets (67, 68). Islets contain a glomerular-like network of fenestrated capillaries that comprise about 8-10% of islet volume. Islet capillary density is estimated to be 10 times higher than that of the exocrine pancreas and is driven by high local production of VEGF-A (69, 70). This rich vascularization and high islet blood flow is autonomously regulated through complex interactions between hormones, metabolites, and the nervous system. While islet blood flow is innately required for and coupled to insulin sensing and release, extensive vasculature also makes the beta-cell uniquely poised for interactions with the immune system.

The dense islet vasculature network facilitates activated immune cell trafficking across the vascular endothelium into the islet (**Figure 1**). This causes a local inflammatory microenvironment which in turn, further increases permeability, facilitating access even for naïve T cells (71). Interestingly, this “open” environment remains, even after reversal of diabetes with anti-CD3 treatment. In addition to naïve T cell infiltration, activated immune cells that are primed locally in the pancreatic lymph nodes (pLNs) can also cross the vascular endothelium. pLNs may contribute to T1D pathogenesis as drainage from the pancreas and local gut regions provides a crossroad for the immune cells traveling between these compartments (67, 72). The interplay between the microbiome, the immune system, and a “leaky gut” has been implicated as a key factor in T1D pathogenesis (73, 74). Toll-like receptors (TLRs) are a family of innate pattern recognition receptors important for microbial clearance by the immune system. Many TLRs signal through the MyD88 adapter protein. NOD mice deficient in MyD88 exhibit microbiota-dependent protection from autoimmunity development (75). Specific manipulation of TLR expression and microbiota composition can further regulate disease progression or prevention (76). Sex-specific autoimmune risk can also be influenced by microbiome manipulation (77–79). With new data unveiling the importance of the microbiome for the gut immune environment and shaping peripheral tolerance, the relationship between pLNs, islet vasculature, and immune cell trafficking is increasingly relevant to beta-cell vulnerability and T1D pathogenesis (80, 81). In addition to facilitating interactions between islet cells and immune cells, the islet vasculature also sensitizes the beta-cell to the damaging effects of circulating proinflammatory cytokines.

**Figure 3:**
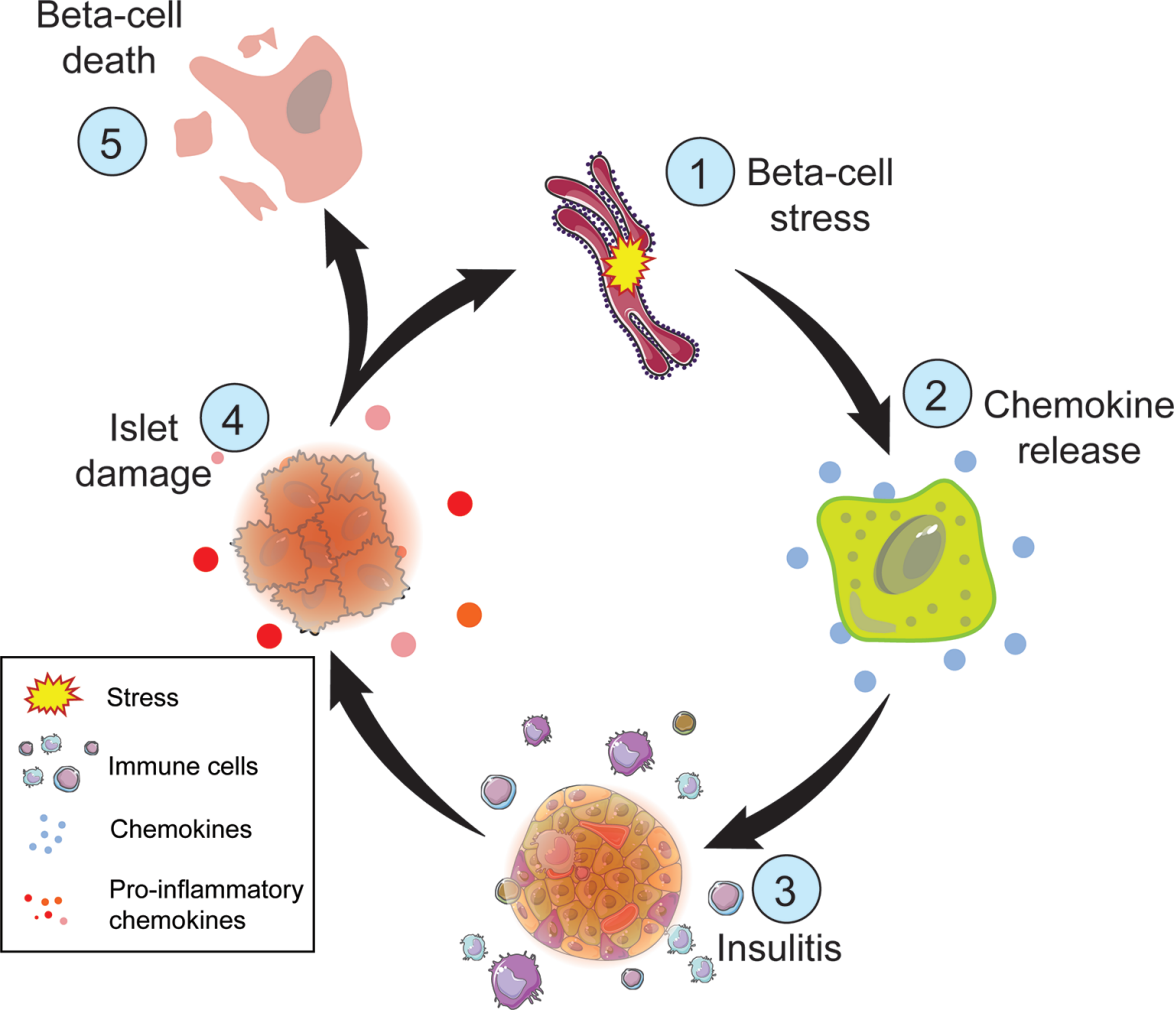
Steps of beta-cell death in T1D. Metabolic demand of nutrient challenge results in ER and oxidative stress (1) followed by chemokine release by the beta-cell (2). Chemokines attract immune cells (3), such as macrophages and T cells, which can damage the islet (4) directly though T cell interactions and indirectly through the release of inflammatory cytokines and reactive oxygen species. Cellular damage exacerbates ER and oxidative stress perpetuating this cycle. The inability to restore cellular homeostasis will result in beta-cell death (5).

Beta-cells are sensitive to cytokine-mediated damage. Cytokines can alter crucial beta-cell characteristics including insulin secretion, mitochondrial function, and intracellular calcium stores (82, 83). Inflammatory cytokines including tumor necrosis factor alpha (TNFα), interferon gamma (IFNγ), and interleukin-1 beta (IL-1β), cause beta-cell dysfunction by impairing ATP production, inducing DNA damage, and promoting apoptosis (**Figure 3**) (84, 85). Proinflammatory cytokine exposure inhibits GSIS due to the limited availability of ATP in both rodent and human islets, as well as in beta-cell lines (86–89). Islet exposure to cytokines triggers nuclear factor κB (NFκB) induction of inducible nitric oxide synthase (iNOS), which increases NO formation in the beta-cell (87). NO has temporal effects on beta-cell responses, as early and transient levels of NO (less than 24 hours) facilitate the repair of cytokine-induced DNA damage by inhibiting the activation of the DNA damage response and preventing the induction of apoptosis (90). However, prolonged exposure to NO can induce beta-cell death due to DNA damage, UPR activation, and decreased mitochondrial oxidation (i.e., ATP production) (87, 91). In cultured islets, pre-exposure treatment with NO inhibitors, such as aminoguanidine, attenuates cytokine-mediated beta-cell death (92). Cytokine-mediated beta-cell death becomes exacerbated in an inflammatory microenvironment in the pancreas, creating a positive feedback loop resulting in more inflammation, stress, vulnerability, and eventually cell death (**Figure 3**). Unfortunately, clinical trials with anti-cytokine therapies such as Anakinra, an IL-1 receptor antagonist, were not efficacious in delaying T1D, suggesting a more complex interaction between cytokines and beta-cells *in vivo* (92). Nonetheless, not every beta-cell is lost in T1D, nor do they all respond negatively to ER and/or oxidative stress, indicating an intrinsic beta-cell heterogeneity in response to disease promoting factors.

## Beta-Cell Heterogeneity

It is challenging to fully understand the response and contribution of the beta-cell to the T1D disease state without understanding beta-cell heterogeneity. Many groups have focused on determining whether different subtypes of beta-cells exist, and if so, how they might differ in functional ways such as proliferative and secretory capacities (93–95). Identifying subpopulations, and then understanding their inter- and intra-islet communication, has uncovered a level of complexity and diversity not previously appreciated. We will briefly explore the recent findings regarding functional and transcriptional heterogeneity of the beta-cell and discuss potential impacts on susceptibility to T1D.

### Functional Diversity

Evidence for functional beta-cell heterogeneity in calcium flux, metabolism, ion channel conductance, and insulin secretion has been appreciated for almost 30 years (96, 97). More recently, this functional diversity has been specifically defined by many research groups into beta-cell subpopulations (**Table 1**). Using novel cell surface markers (ST8SIA1 and CD9) identified by immunizing mice with human islets, four human beta-cell subtypes with unique basal and GSIS responses were defined as β1-4 (93). All subtypes contain insulin granules but exhibit variable functionality and abundance; β1 is the most abundant and glucose responsive, while β4 is the rarest and least responsive, with highest basal insulin secretion. Interestingly there are no correlations found between subtype ratios and sex, age, or obesity, but subtype abundance is altered and much more variable in Type 2 diabetes (T2D). For example, β3 and β4 subsets are unusually overrepresented in T2D islets, but abundance of these two subsets varied much more than in control non-diabetic tissue. It is unknown if β1-4 cells exist in unique patterns before or after T1D diagnosis and disease progression.

**Table 1:** Selected pancreatic beta-cell subtypes. Heterogeneity in beta-cell response has led to the identification of beta-cell subtypes. These subtypes may vary in spatial location within the islet, speed of response to a stimulus, and secretory capacity. The names of beta-cell subtypes, description of characteristics, and whether they were identified in mouse or human pancreata are defined below.

Name of Subtype	Description of Characteristics	Mouse or Human	Reference
β1	Highest GSIS, least abundant in T2D tissue	Human	Dorrell et al., 2016 (63)
β2	CD9^+^, ST8SIA1^-^, stimulation index second highest after β1	Human	
β3	CD9^-^, ST8SIA1^+^, increased in T2D	Human	
β4	Lowest GSIS, high basal secretion, increased in T2D	Human	
Hub	Pacemaker, responds quickly to calcium influx, makes up 1-10% of beta-cell mass	Both	Johnston et al., 2016 (98)
Virgin	Transcriptionally and functionally immature (UCN^-^), located at islet periphery, incapable of glucosensing	Both	Van der Meulen et al., 2017 (65)
Flattop+	Mature, functional secretory granules, increased with high fat diet	Mouse	Bader et al., 2016 (69)
Flattop-	Immature, highly proliferative, Wnt^+^ (Become flattop^+^)	Mouse	
Top	Present in non-T1D setting, glucose responsive, express maturity markers	Mouse	Rui et al., 2017 (99)
Bottom	Population appears in a T1D environment, resistant to immune killing, unresponsive to glucose, express stemness markers	Mouse	
First-Responder	First to respond to calcium influx, other beta-cell response based on distance from these	Mouse	Kravets et al., 2020 (70)
Extreme	High levels of proinsulin and ribosomes, low insulin protein content, increased in *db/db* mice	Mouse	Farack et al., 2019 (78)

In 2016, Johnston et al. discovered specialized beta-cells they termed “hub” cells that exert disproportionate control over blood glucose (94). Hub cells, comprising 1-10% of islet beta-cell mass, are metabolically active, exhibit evidence of transcriptional immaturity (low or absent Pdx1 and Nkx6.1 transcription factor levels), and are hypothesized to act as a pacemaker within the islet. Supporting this model, calcium tracing showed that surrounding cells, termed “followers”, respond to glucose stimulation slightly after the hub cell. Using optogenetic and pharmacological techniques, silencing hubs caused desynchrony in the calcium-induced response of beta-cells. The Huising group reported on a beta-cell subtype located in the islet periphery that is both transcriptionally and functionally immature, called the “virgin” beta-cell (95). Virgin cells lack the maturity marker urocortin 3 (UCN3) and are incapable of sensing glucose or proper calcium influx. Using lineage tracing, they found that these cells are transdifferentiation intermediates between alpha- and beta-cells. This work defined a “neogenic niche” of new beta-cells originating from an alpha-cell lineage, establishing a plasticity between these cell types that had also been suggested by models of extreme beta-cell loss (100). The hub versus virgin subpopulations can be compared to flattop^+^ and flattop^–^ populations, where this novel marker differentiates between a mature, functional beta-cell population (flattop^+^) and an immature and highly proliferative one (flattop^-^) (101).

In 2020, the Benninger lab defined yet another subpopulation that is functionally distinct from those previously described (102). These cells are termed “first-responder” beta-cells, as defined by calcium dynamics. They found that first-phase response time of beta-cells is spatially organized and dependent on the physical distance from the first-responder cell. How these sub-populations of cells as defined by these specific markers may be unique or overlapping remains to be determined.

### Transcriptional Diversity

The implementation of single-cell transcriptomics has allowed exploration of beta-cell heterogeneity on a scale that was not previously possible. The Kubicek lab published the first application of single-cell transcriptomics to human islet cells in 2016 (103); while the number of beta-cells identified was extremely low, they showed transcriptome heterogeneity for genes such as *DLK1*, a T1D associated gene by GWAS (Genome-Wide Association Study) which will be discussed later in more detail. That same year the Kaestner lab published single-cell mass cytometric analysis of human islets and found heterogeneity in markers including Ki67, identifying four distinct beta-cell subpopulations (104). Several groups have since published large-scale single-cell transcriptome analyses of human and mouse beta-cells and identified unique subpopulations. These populations display differential levels of various beta-cell characteristics including maturity (UCN3), aging (IGF1R), and ER stress response (SRXN1, SQSTM1) (105, 106). Genes involved in ER/oxidative stress have been some of the strongest distinguishers of subpopulation clusters in various studies, with ER stress markers also correlating to proliferation and reduced beta-cell function (107–109). As discussed earlier, beta-cell ER stress is extremely relevant to an understanding of T1D contribution and response. Perhaps certain populations of proliferative-ER stressed beta-cells are the first to be lost during T1D pathogenesis.

For years, technical hurdles to interrogating mRNA in islets due to the digestive enzymes present in the surrounding exocrine pancreas limited our understanding of transcriptional dynamics and heterogeneity in the beta-cell. Dr. Shalev Itzkovitz at the Weitzmann Institute developed methods to visualize the dynamics of beta-cell mRNAs. His group designed an optimized single-molecule FISH (fluorescence *in situ* hybridization) protocol that allowed for assessment of transcriptional heterogeneity within the beta-cell population (110). This method revealed a subpopulation of “extreme” beta-cells that contain high levels of *insulin* and secretory factor mRNAs (*IAPP*, *ChgA*), but interestingly, low levels of insulin protein. The investigators suggested this may impart a specialization for basal insulin secretion. Additionally, beta-cell mRNAs displayed a uniquely polarized pattern, with elevated *Insulin* mRNA concentration in apical ER enriched compartments of the cell. The ratio of extreme beta-cells was increased in *db/db* diabetic mice, potentially facilitating increased requirements for basal insulin. This work gives rise to another unappreciated aspect of beta-cell heterogeneity: transcriptional heterogeneity. Future studies are needed to explore the proportion of extreme beta-cells in T1D and how this type of transcriptional variability may affect vulnerability to autoimmune recognition and attack.

The relationship between these different beta-cell subpopulations is still being defined, as the markers discussed above may represent unique or overlapping populations. For example, each of these functional and transcriptional subpopulations represents a unique niche that may have specific susceptibilities or contributions to T1D. Is a hub cell the same as a β1 or flattop^+^ cell? What are the differences and are they physiologically relevant? What is the relationship between these cell types and how do those relationships change with age and nutrition state? Understanding the relationship between functionality, heterogeneity, and vulnerability will provide a deeper understanding of T1D etiology, potentially setting the stage for more effective therapeutic strategies.

## Beta-Cell Contributions to Inflammation

Therapeutic strategies for preventing or treating T1D have historically focused on modulating the immune response to the beta-cell. Emerging strategies that instead focus on beta-cell dysfunction through manipulation of ER, oxidative, or cytokine-induced cell stressors may prove to be beneficial, as the beta-cell itself actively contributes to inflammatory responses in T1D. The following section will discuss beta-cell contributions to the T1D inflammatory environment, which may represent optimal targets for future combinatorial therapies.

### Chemokine Production

Chemokines are a family of small molecules involved in lymphoid physiology, pathology, and hemopoietic cell migration (111, 112). Chemokines can be broadly separated into two categories: constitutive and inducible (113). Constitutive chemokines perform homeostatic functions involving non-inflammatory leukocyte trafficking, while inducible chemokines are produced in response to inflammation to recruit activated leukocytes to the sites of damage or stress (114). Multiple chemokines, detailed below, are secreted by the beta-cell and contribute to immune infiltration into the islet (**Figure 2I**). These secreted factors make the beta-cell a target for immune invasion and destruction.

#### CCL2

The chemokine C-C ligand 2 (CCL2) also known as monotype chemoattractant protein (MCP)-1 is an inducible chemokine involved in monocyte, NK cell, and T cell recruitment during inflammation (115–117). Human and NOD islets cultured with proinflammatory cytokines IL-1β and IFNγ can induce CCL2 production (118–120). Beta-cells express *Ccl2* in an NF-κB−dependent manner and can be induced *in vivo* by environmental triggers such as viral infections leading to inflammation and macrophage recruitment (121, 122). Macrophages are the first and most abundant immune cell to infiltrate the islet during the progression of T1D in NOD mice and have also been identified in islets from patients with recent-onset T1D (115, 123, 124). CCL2 may be responsible for this influx in macrophages, as transgenic overexpression of *Ccl2* in murine beta-cells results in increased monocyte recruitment, insulitis, and islet destruction (115). Binding of CCL2 to its receptor C-C chemokine receptor-2 (CCR2) in macrophages leads to the production of proinflammatory cytokines and chemokines such as TNFα, IL-1β, IL-12, and CXCL10 to exacerbate the inflammatory environment of the islet (125). Prolonged exposure to proinflammatory cytokines leads to ER stress, oxidative stress, and cell death (115) (**Figure 3**).

#### CCL5

CC ligand 5 (CCL5) also called RANTES (regulated on activation, normal T cell expressed and secreted) is a chemoattractant for T cells, eosinophils, and monocytes involved in inflammatory responses. CCL5 has been measured in rodent islets and from cell sorted beta-cells in response to inflammatory cytokines TNFα, IL-1β, and IFNγ (126, 127). Increased expression of CCR5, one of the cognate receptors for CCL5, was detected on T cells from patients with T1D and NOD mice (128, 129). Blocking CCR5 using neutralizing antibodies in 2-month-old NOD mice (after islet infiltration, but before overt diabetes) inhibits future immune infiltration and prevents development of diabetes (126).

#### CXCL10

*C-X-C* motif chemokine ligand 10 (CXCL10) also called IP-10 (IFNγ-induced protein 10) is a chemokine secreted by many cell types including monocytes, neutrophils, and endothelial cells (130, 131). CXCL10 is increased in the serum and tissues of patients with various autoimmune diseases including T1D (99, 132). Human islets, murine islets, and NIT-1 NOD beta-cells secrete CXCL10 when cultured with pro-inflammatory cytokines IL-1β and IFNγ (127). CXCL10 binds the seven transmembrane G protein coupled receptor CXC receptor 3 (CXCR3), expressed on both immune and non-immune cells (133, 134). In lymphocytes, CXCR3 mediates chemotaxis, while in non-lymphocytes CXCR3 regulates tissue repair, proliferation, and angiogenesis (135, 136). Mice lacking CXCR3 and infected with lymphocytic choriomeningitis virus-WE strain (LCMV-WE), an established model to study T1D, exhibited a delay in insulitis, while overexpressing CXCL10 in mouse islets accelerated LCMV-induced diabetes (127, 137, 138). As predicted, using neutralizing antibodies to block CXCL10 also decreased T cell trafficking to the islet and abrogated diabetes development (139–141). In culture, the NIT-1 NOD beta-cell line was found to secrete CXCL10 in response to inflammatory cytokines IL-1β, TNFα, and IFNγ (127). These data suggest that elevated CXCL10 secretion by the beta-cell may occur early in T1D progression. CXCL10 not only contributes to immune cell recruitment but is also directly toxic to beta-cells (142). In addition to CXCR3, CXCL10 also binds Toll-like receptor-4 (TLR4), a pattern recognition receptor involved in the immune response to microbial pathogens (143). The CXCL10:TLR4 signaling pathway in beta-cells leads to cleavage and translocation of activated protein activated kinase 2 (PAK-2) into the nucleus, contributing to apoptotic signaling within the cell (144, 145). Islets from C57BL/6 *Tlr4*^-/-^ knockout mice are protected against CXCL10-induced damage. Therefore, CXCL10 released by the beta-cell contributes to cell death by attracting activated immune cells and inducing apoptosis within the beta-cell.

Beta-cells produce proinflammatory CCL2, CCL5, and CXCL10 chemokines when exposed to inflammatory conditions or environmental triggers and can perpetuate the recruitment of immune cells to initiate insulitis. Once present, these immune cells can damage the beta-cell by synthesizing ROS, proinflammatory cytokines, and expressing receptors that can directly mediate beta-cell death (**Figure 2**).

### Beta-Cell Promotion of Cellular Death

Of the infiltrating cells causing insulitis in T1D, T cells are the major destroyer of beta-cells, with both CD4 and CD8 T cells being required to effectively transfer disease (146–148). CD4 and CD8 T cells have different roles in disease development (149). When activated, CD4 T cells or T “helper” cells influence the activation of surrounding immune cells through the production of pro- or anti-inflammatory cytokines (149). Human CD4 T cells conventionally recognize peptides presented on HLA-II molecules expressed by antigen-presenting cells (APCs), but the expression of HLA-II has also been detected on beta-cells from patients with T1D (150). CD8 T cells recognize peptides presented on MHC-I on mouse cells and HLA-I on human cells (**Figure 2D**). Islets biopsied from patients with T1D displayed HLA-I hyperexpression, which warrants their susceptibility to CD8 T cell-mediated destruction (151). The activation of CD8 T cells leads to the differentiation of CD8 T cells to become cytolytic T lymphocytes (CTLs) resulting in the directed release of cytotoxic cytokines, cytolytic granules, and Fas ligand (FasL)-mediated death of the target cell. Beta-cells from diabetic patients not only express HLA-I/II molecules, but also the Fas receptor (CD95/Apo-1) (152–154) (**Figure 2J**). Fas/FasL signaling is suggested to play a role in T1D pathology as NOD mice deficient in Fas do not develop inflammation or diabetes (155). Fas-deficient mice are also protected against adoptive transfer of splenocytes from diabetic NOD mice. In rodent and human islets, the expression of Fas receptor in beta-cells is induced by proinflammatory cytokines IL-1α, IL-1β, IFNγ, and the upregulation of iNOS, as sequestering NO in the beta-cell decreases Fas expression (156, 157). Fas/FasL signaling in the beta-cell leads to apoptosis *via* the activation of caspase 8 and the mitochondrial pathway of apoptosis (158). Beta-cell-derived proinflammatory chemokines, HLA-I/II (or MHC-I/II) molecules, and Fas/FasL receptors can perpetuate T1D disease progression by promoting immune cell recruitment, T cell activation, and subsequent beta-cell destruction. Since autoantibodies can be detected in circulation for years prior to disease onset (159) and patients from the Medalist study (discussed below) retain a portion of insulin-secreting beta-cells, these observations provide evidence that at least some beta-cell populations may possess mechanisms to evade the immune response.

## The T1D Beta-Cell

In addition to the intrinsic, “baseline” heterogeneity of beta-cells, heterogeneity of disease progression within islets from individual patients, and heterogeneity of disease progression amongst patients with T1D are becoming apparent through longitudinal clinical studies and new analytical techniques examining T1D animal models. The Joslin Medalist Study of T1D patients with disease duration of 50 years or longer revealed that some insulin producing beta-cells persist, ostensibly even after years in a chronic inflammatory environment (160). This highlights that some level of heterogeneity is present in the T1D islet, supporting that certain beta-cell populations may be protected from autoimmune destruction. The expansion of single-cell transcriptomics has contributed to our understanding of cell populations dynamics, but whether it be mouse or human, almost all published studies have used either healthy or T2D islets.

Exciting work in the past few years has given rise to the idea that disease-specific beta-cell heterogeneity may arise during T1D progression, with certain populations that are more vulnerable than others to autoimmune-mediated death. The Herold lab was one of the first to identify distinct cell populations in T1D with their discovery of a low granularity beta-cell population termed “bottom” cells in the NOD mouse model (161) (**Table 1**). They found that this non-glucose-responsive population emerges prior to hyperglycemia and immune infiltration and expands over time, comprising over 50% of the beta-cell population by 12 weeks of age. The bottom cells express “stemness” markers and were found to be less sensitive to treatment with cytokines and immune infiltrates compared to their “top” counterparts, suggesting they may evade immune attack (161). As we continue to understand disease etiology more deeply, these resistant populations may provide a novel target for treatment.

The discovery of distinct T1D endotypes associated with age of diagnosis has recently contributed to our knowledge of the T1D beta-cell (162). Using immunohistochemical analysis of pancreas samples from patients diagnosed under the age of 30, Leete et al. found a distinct pattern of insulin/proinsulin localization in the beta-cell that is not present in non-T1D controls. Specifically, they found high insulin/proinsulin colocalization in patients who were diagnosed under 13, and even more consistently in patients diagnosed before 7 years of age. Similar subtypes had been described regarding insulitis, with two discrete histological profiles associating strongly with age of diagnosis (163, 164). The authors postulate that discovery of these histologically distinct phenotypes points to disease endotypes that could even be described as T1DE1 and T1DE2 and may require different immunotherapeutic options based on age of diagnosis. While this work is not necessarily beta-cell specific, the heterogenous nature of disease that the field continues to uncover further points to the importance of understanding beta-cell heterogeneity and response to autoimmunity. We propose that an understanding of beta-cell dynamics prior to, during, and after immune-cell infiltration in T1D will be vital to development of therapies that can not only combat T1D development, but perhaps even precede and bypass it.

## T1D-Associated Beta-Cell SNPs

T1D pathogenesis involves both genetic and environmental triggers and susceptibilities. Thus, examining genetic associations that link specific loci to T1D vulnerability has been a major area of research focus. Surprisingly, the Wellcome Trust Case Control Consortium (WTCCC) established GWAS found relatively few novel risk loci for T1D. It was not until the T1D Genetics Consortium (T1DGC) conducted a meta-analysis that approximately 41 distinct susceptibility loci were identified (165). Fine mapping of these loci using ImmunoChIP established credible sets of single nucleotide polymorphisms (SNPs), most of which are found in non-coding DNA regulatory regions, including tissue-specific enhancers (166). Most of our understanding of the identified SNPs has been centered around the *HLA* loci that are strongly associated with the disease and T cell autoreactivity. While these studies of the immune arm of pathogenesis are invaluable, not only do SNPs in the *INSULIN* (*INS*) gene remain one of the highest risks (167), but approximately 60% of all T1D susceptibility genes are expressed in the islet (168). These data further support the concept that the beta-cell has a larger role in its own destruction than previously appreciated, and that genetic susceptibility is not solely based on the status of the immune system. Below we describe beta-cell-associated SNPs and what is known thus far about their T1D implications.

### Insulin

As mentioned above, after the *HLA* locus, the 5’ upstream region of the *INS* locus is the genomic region with the strongest association with T1D risk (167). Specifically, it is the INS-VNTR (variable number tandem repeat) locus that confers susceptibility differences. The VNTR alleles are defined by two classes: class I (26-63 repeats) and class III (140-200 repeats). The shorter class I VNTRs confer a 2-5-fold increase in T1D risk while the longer class III allele is protective against T1D, this is thought to be due to effects on proinsulin expression in the thymus (169). Class III VNTRs are associated with increased *INSULIN* transcription in the thymus during induction of central immune tolerance. The authors proposed that these increased thymic insulin levels may promote negative selection of insulin-specific T cells, ultimately leading to a protective effect on T1D susceptibility. Class I VNTRs result in decreased *INSULIN* transcription in the thymus and potentially allow insulin-specific autoreactive T cells to escape from the thymus due to defects in central tolerance and negative selection. This class I versus III allele-specific mechanism illustrates the complexity of T1D risk, and while this susceptibility locus can absolutely be seen as a beta-cell associated SNP, the proposed mechanisms that have been defined thus far are still largely immune system focused. So, despite the 100 years since the discovery of insulin, large gaps in our understanding of how it is involved in the response of the beta-cell itself in T1D remain.

### GLIS3

Due to the scarcity of studies of beta-cell contributions to T1D, many associations have been made between beta-cell death and failure in T1D and T2D. Remarkably, GWAS indicates there are very few susceptibility loci associated with both maladies. Variations of the Kruppel-like zinc finger transcription factor *GLIS3* are one of the few that have been strongly associated with both T1 and T2D (170). Also a known MODY (Maturity Onset Diabetes of the Young) gene, *GLIS3* is expressed predominantly in the pancreas, thyroid, and kidney. While there have been some discrepancies in the literature regarding the exact timing of expression, in GLIS3-EGFP knock-in mice, GLIS3 mainly co-expresses with Sox9 in bipotent pancreatic islet progenitor cells and is absent from the acinar progenitors at embryonic day (E)13.5. This pattern correlates with its importance in development of the pancreatic endocrine lineage and seeming negligibility for the exocrine portion of the pancreas. The association between GLIS3 and T1D was first identified in European populations but has more recently been recapitulated in a Pakistani cohort (171, 172). Interestingly, a GLIS3 variant (A908V) is associated with T1D resistance in Japanese patients (173). Considering the thymic expression of GLIS3, the authors propose that perhaps this variant induces central or peripheral immune tolerance more efficiently than the wild-type variant, but more studies are needed to understand this mechanism. Regarding the molecular mechanisms that underlie the associations of GLIS3 and diabetes, little was known until multiple groups independently generated both global and beta-cell-specific *GLIS3* knockout models (174–176). Not only do *GLIS3*^-/-^ mice die within the first few days of life, but their islet area is approximately 15% that of littermate controls (177). Insulin production was reduced by 80%, making it difficult to assess GSIS in these mice. These groups also found that the endocrine progenitor gene, *Ngn3*, is a GLIS3 target, and that GLIS3 physically and functionally interacts with the beta-cell transcription factor Pdx1 to regulate *insulin* transcription. Interestingly, GLIS3 overexpression leads to an upregulation of *Ngn3* mRNA in ductal cells, further supporting the role of GLIS3 in pancreatic islet progenitor specification. Pancreatic progenitors, as well as the adult acinar compartment, seem to be unperturbed in GLIS3 knockouts showing an islet progenitor specificity to its role during development. These mechanisms do not tie directly to T1D association but understanding the role of GLIS3 in beta-cell identity may help unveil the mechanisms involved in both T1 and T2D disease susceptibility.

### CLEC16A

C-type lectin domain family 16, member A (*CLEC16a*) is a gene locus associated with T1D, multiple sclerosis, and adrenal dysfunction (98, 178, 179). Though genetic associations have been long established, until the work of Soleimanpour et al., a molecular basis for how CLEC16A might increase T1D was unknown. Interestingly, these investigators found that mouse Clec16a interacts with Nrdp1 (an E3 ubiquitin ligase) and has roles in normal GSIS in the beta-cell (180). Pancreatic *Clec16a* deletion causes reduced ATP levels and mitochondrial oxygen consumption, establishing the factor as a novel regulator of beta-cell mitophagy. Additionally, patients with the T1D-associated SNP in the *CLEC16A* gene exhibit reductions in CLEC16A expression and perturbed insulin secretion.

These observations of impaired insulin and glucose homeostasis, along with ER-stress in their mouse model, are some of the few providing insight into the non-immune related mechanisms of T1D (180). ER-stress and perturbations in first-phase insulin release are among the earliest signs of T1D, predating immune infiltration and insulitis (181, 182). The role of Clec16a in these processes not only highlights its crucial role in beta-cell function, but also establishes it as a potential player in the first steps of beta-cell vulnerability in T1D.

### DLK1

Delta-like 1 (DLK1), also known as DLL1 or Pref-1 (preadipocyte factor 1), is a transmembrane protein that belongs to the Delta-Notch signaling family. Both mouse and human *Dlk1* are known to be subject to genomic imprinting, and Dlk1 is paternally inherited, with the maternal gene being silenced during development. This becomes potentially interesting considering the sexual discordance in inheritance risk in T1DM, as risk of transmission to offspring is 1.7 fold higher from diabetic fathers than mothers (183). However, Wurst et al. found that, in the case of gestational diabetes mellitus (GDM), serum Dlk1 levels were not significantly different between diabetic and control patients (184). Mouse Dlk1 is expressed highly and ubiquitously during development in the embryo and placenta, starting around E11.5, but becomes downregulated in most adult tissues. Adult Dlk1 expression becomes restricted to the beta-cell, bone marrow, pituitary, and adrenal glands. Some evidence suggests that Dlk1 may help undifferentiated cells maintain their pluripotent state, working as a growth factor to maintain proliferation. In preadipocytes, Dlk1 must be downregulated for differentiation to occur (185). Rodent models have remained somewhat controversial, as *Dlk1* null mice have partially penetrant neonatal lethality and complex adult and developmental phenotypes, yet conditional loss of function models in various tissues using floxed mice failed to recapitulate null phenotypes (186, 187). Dlk1 beta-cell knockout mice were found to be fully viable with normal islet architecture up to six weeks of age, though glycemic control was not assessed. More thorough analyses of Dlk1 in the beta-cell, including insulin secretion and glucose tolerance, are required to fully understand if and how it may contribute to both function and potentially pathogenesis of T1D.

The genetic basis of T1D pathogenesis is complicated and still poorly understood. A majority of these studies have been conducted using data exclusively from Caucasian patients, and inclusion of multi-ethnic populations is required for a more complete and accurate understanding of genetic variants. The few studies using African-ancestry participants have already yielded unique haplotypes and signatures (188, 189). Additionally, as mentioned above, non-coding DNA regulatory regions make up a majority of T1D associated SNPs, which suggests that genetic variation may be impacting regulatory functions rather than gene-coding abilities (165, 166). Gene expression can be controlled *via* long-range interactions, with regulatory elements impacting genes that are hundreds of kilobases away. Understanding these potential interactions requires employment of techniques such as chromatin conformation capture, building a more complete picture of how these SNPs may regulate distant genes through physical contact with non-adjacent promoters. Recently, the use of chromatin-accessibility quantitative trait loci (caQTL) and fine mapping analysis expanded the genetic variants and loci associated with T1D and provided novel molecular targets to investigate (190). Whether it be immune or beta-cell related, understanding these “true” gene targets is a vital steppingstone in leveraging this genetic information to develop diagnostic and therapeutic solutions to T1D.

## Clinical Applications: Beta-Cell Directed Therapeutics

Therapeutic strategies for preventing T1D in high-risk patients have often focused on modulating the immune response to the beta-cell. Newer strategies include methods that focus on the beta cell: reduction of beta-cell dysfunction through the manipulation of ER, oxidative, or cytokine induced cell death. Additionally, functional beta-cell mass replacement strategies through alternative sources, such as stem cells, are being exploited in the field. These strategies, however, will likely have limited clinical utility until autoimmune destruction of the beta-cell replacement can be avoided. Therefore, combinatorial therapeutic programs will likely be required to truly prevent or reverse T1D. Here we describe the state of a few current beta-cell focused therapeutics.

### Modulation of ER-Induced Beta-Cell Death

Development of compounds targeting ER-stress pathways are being explored to prevent beta-cell death in early onset T1D. The three UPR sensors PERK, ATF6, and IRE1α regulate apoptosis and thus make a promising target for reducing ER stress and subsequent death in the beta-cell (191). Tauroursdoxycholic acid (TUDCA), a naturally occurring bile acid, can reduce ER-stress by inhibiting the dissociation of BiP from PERK, preventing cell death (192). In a multiple low-dose STZ C57BL/6 mouse model of beta-cell death, TUDCA improved glucose tolerance, increased beta-cell mass, and improved glycemia compared to control diabetic mice (193). The benefits of TUDCA and other UPR chaperones continue to be investigated for their ability to prevent ER-stress induced apoptosis in T1D (194).

An ongoing clinical trial using imatinib mesylate (brand name Gleevec), a tyrosine kinase inhibitor, shows promising results in targeting beta-cell ER stress (195). The efficacy of imatinib for the treatment of various immune-mediated diseases is currently being tested. Initially found to abrogate type 2 diabetes in *db/db* mice, imatinib treatment in the NOD mouse was able to reverse autoimmune diabetes (196, 197). By blunting IRE1α RNase hyperactivity, imatinib reduces beta-cell apoptosis and preserves physiological function. In humans, a clinical trial found imatinib preserved beta-cell function at 12 months in adults with recent-onset T1D (195). Ongoing studies will investigate dose and duration of therapy as well as safety and efficacy for use in children.

### Targeting Oxidative Stress

Considering the major role of oxidative stress in T1D pathogenesis, therapies designed to improve antioxidant defenses in beta-cells are another promising avenue for clinical use. Thioredoxin interacting protein (TXNIP), a thioredoxin (TRX) inhibitor of the peroxiredoxin/thioredoxin detoxification pathway has demonstrated clinical potential in both animal models and initial clinical trials (198). The binding of TXNIP to TRX promotes oxidative stress by preventing peroxide clearance. TXNIP is elevated in patients with T1D and T2D (199). *In vivo* overexpression of TXNIP in mouse beta-cells induces apoptosis, while inhibition is protective against STZ-induced diabetes (200). Anti-diabetic agents including insulin and metformin, were found to augment TXNIP degradation through activation of adenosine monophosphate activated protein kinase (AMPK), supporting the idea that TXNIP may be a viable clinical target (201). Verapamil, which blocks voltage-gated calcium channels, decreased TXNIP and enhanced beta-cell survival in both human and rodent islets (202). A clinical trial in which Verapamil was administered (along with insulin therapy) promoted patient beta-cell function and lowered exogenous insulin requirements (203). TXNIP is expressed in multiple cell types throughout the body and additional clinical trials (NCT04545151, NCT04233034) are ongoing to explore the efficacy of TXNIP inhibition in protecting beta-cells and affecting autoimmune responses in patients with T1D.

For both imatinib, an FDA approved anti-leukemia drug, and verapamil, a widely used anti-hypertensive, the ability to repurpose drugs already on the market and with established safety profiles for T1D is an attractive way to expedite the often lengthy and costly process of bringing drugs to market.

### Stem Cell Derived Beta-Cells and Emerging Technologies

The development of human stem cells for clinical use may provide a long-term solution to T1D without the challenge of organ shortage and HLA mismatch. While for years now, stem cell-derived insulin-producing cells can be generated and studied in the lab, improvements in cell viability, identity, and reproducibility may be needed before they can be applied as a safe and affordable therapy (204). Beta-cell differentiation from multiple cell sources have been attempted, the most promising of which seem to be induced pluripotent stem cell (iPSC)-derived beta-cells (205). Clinical trials are in progress investigating the efficacy of treating T1D with one version of iPSC derived cells designed by Viacyte, Inc. and CRISPR (Clustered Regularly Interspaced Short Palindromic Repeat) Therapeutics (NCT03163511).

Combining iPSC-derived cells with CRISPR gene editing technology allows the potential to correct monoallelic mutations in genes, such as those that cause MODY diabetes. Optimal for T1D treatment, some therapies are also focused on creating “stealthy” beta-cells and islets, that can be specifically engineered to evade immune recognition. One gene of interest is *renalase* (*Rnls)*, which encodes for an FAD-dependent amine oxidase enzyme that was identified in NIT-1 cells. *Rnls* deletion elicits beta-cell protection against autoimmune attack. NIT-1 cells carrying the *Rnls* mutation improved graft survival when transplanted into diabetic NOD mice (206). Using CRISPR, human iPSCs were generated lacking *RNLS*. These *RNLS^-/-^* iPSCs could be successfully differentiated and exhibited normal insulin secretion *in vitro*. Thus far, the *in vivo* function of these cells has not been reported. Similarly, some groups have recently generated iPSC lines that are “hypoimmune” by inactivating MHC class I and II genes and overexpressing protective marker CD47 (207, 208). These stem cells evade immune rejection in fully competent recipients, while maintaining their pluripotency. Hypoimmune stem cells have been used to treat pulmonary and cardiovascular disease and have major implications for universal transplantation (209). The widespread availability of techniques such as single-cell RNA sequencing can bolster the design of iPSC derived beta-cells by identifying the gene expression repertoire needed to obtain the appropriate distribution of cell types within the islet (210).

An exciting new technology that may aid in our understanding of the T1D beta-cell is Patch-Seq, a powerful method that can link single-cell transcriptomes with electrophysiology measurements (211). Groups using this technique in the beta-cell may be able to uncover various levels of beta-cell heterogeneity and link it to functionality in both healthy and T1D contexts. The Yoshihara group is also working on combining concepts with their work reproducing disease with 3D organoids engineered to model immune invasion (212). Overexpression of PD-L1 in human islet organoids was able to protect xenografts from immune invasion and restore glucose homeostasis for 50 days in immune competent mice. Similarly, the Melero-Martin group has used a combinatorial approach to incorporate the importance of islet vasculature in their studies. Their “vascular organoids” include microvessels that become perfused during transplantation and even reduce the islet requirement for transplantation, highlighting the importance of vascular cell types for ideal glucose regulation (213). More combinatorial therapies should be explored as we continue to uncover the nuances of the beta-cell and immunological interface of T1D pathology.

## Conclusions

We have highlighted the crosstalk between pancreatic beta-cells and the immune system in T1D and potential mechanisms by which innate beta-cell characteristics contribute to T1D initiation and progression. Beta-cells display an increased vulnerability to destruction and can also perpetuate inflammatory and autoimmune responses in a destructive positive feedback loop. Despite recent advances in technologies such as single-cell sequencing and the optimization of differentiation protocols for stem cell-derived beta-cells, we still have an incomplete understanding regarding the dynamics of beta-cell biology in T1D. In particular, the relationship between the transcription factors involved in beta-cell heterogeneity that can influence immune evasion versus immune susceptibility need to be further defined. Beta-cell vulnerability to oxidative stress needs to be further explored, as redox-dependent signaling pathways influence numerous facets of beta-cell biology including the differentiation of beta-cell subtypes in T1D. Many of the aforementioned emerging technologies have been examined in T2D in the islet but have not been studied in T1D. Despite the challenges, more studies of human islets before, during, and after autoimmunity in T1D should be performed to improve our understanding of beta-cells that can resist immune destruction, and therefore our ability to design more effective treatments. Could an exploitation of beta-cell populations that are less vulnerable prevent or delay T1D onset? Perhaps as we understand these “resistant” populations more fully, therapies can target and pharmacologically expand them.

## Author Contributions

ET, KB, HT, CH, and RB outlined, wrote, and edited the manuscript. All authors contributed to the article and approved the submitted version.

## Conflict of Interest

The authors declare that the research was conducted in the absence of any commercial or financial relationships that could be construed as a potential conflict of interest.

## Publisher’s Note

All claims expressed in this article are solely those of the authors and do not necessarily represent those of their affiliated organizations, or those of the publisher, the editors and the reviewers. Any product that may be evaluated in this article, or claim that may be made by its manufacturer, is not guaranteed or endorsed by the publisher.

## Glossary

**Table:**

ATF6	activating transcription factor 6	
AMPK	adenosine monophosphate activated protein kinase	
APC	antigen-presenting cell	
BiP	binding immunoglobulin protein	
CCR2	C-C chemokine receptor-2	
CCL2	C-C ligand 2	
JNK	c-Jun N-terminal Kinase	
Clec16A	C-type lectin domain family 16, member A	
CXCL10	*C-X-C* motif chemokine ligand 10	
Ca^2+^	calcium	
GPx	glutathione peroxidase	
CCL5	CC ligand 5	
ChgA	chromogranin-A	
CRISPR	Clustered Regularly Interspaced Short Palindromic Repeat	
CXCR3	CXC receptor 3	
DLK1	Delta-like 1	
LCMV-WE	diabetogenic lymphocytic choriomeningitis virus-WE strain	
E	embryonic day	
ER	endoplasmic reticulum	
FasL	Fas ligand	
FISH	fluorescence *in situ* hybridization	
GDM	gestational diabetes mellitus	
Glut2	glucose transporter 2	
GSIS	glucose-stimulated insulin secretion	
Gad65	glutamic acid decarboxylase 65	
GWAS	Genome-Wide Association Study	
HLA	human leukocyte antigen	
HIPs	hybrid insulin peptides	
H_2_O_2_	hydrogen peroxide	
iPSC	induced pluripotent stem cell	
iNOS	inducible nitric oxide	
IRE1a	inositol-requiring enzyme 1 alpha	
*INS*	*INSULIN*	
IFNγ	interferon gamma	
IL-1β	interleukin-1 beta	
IP-10	IFNγ inducible protein 10	
IAPP	islet amyloid polypeptide	
LADA	latent autoimmune diabetes in adults	
MHC	major histocompatibility chain	
MODY	maturity onset diabetes of the young	
MCP)-1	monotype chemoattractant protein	
NOD	non-obese diabetic	
NFκB	nuclear factor κB	
Pdx1	pancreatic and duodenal homeobox 1	
pLNs	pancreatic lymph nodes	
PAD	peptidyl arginine deiminase	
PTM	post-translational modification	
Pref-1	preadipocyte factor 1	
PAK-2	protein activated kinase 2	
PERK	protein kinase RNA-like endoplasmic reticulum kinase	
RANTES	regulated on activation, normal T cell expressed and secreted	
ROS	reactive oxygen species	
*Rnls*	renalase	
SNPs	single nucleotide polymorphisms	
SOD	superoxide dismutase	
T1DGC	T1D Genetics Consortium	
TUDCA	tauroursdoxycholic acid	
TRX	thioredoxin	
TXNIP	thioredoxin interacting protein	
tTG2	tissue transglutaminase 2	
TLR4	Toll like receptor-4	
TNFα	tumor necrosis factor alpha	
T1D	Type 1 diabetes	
T2D	Type 2 diabetes	
UPR	unfolded protein response	
UCN-3	urocortin-3	
VNTR	variable number tandem repeat	
WTCCC	Wellcome Trust Case Control Consortium	
